# Health reforms across the world: are they heading in the same direction, and how much change can we expect?

**DOI:** 10.1007/s43999-023-00024-8

**Published:** 2023-06-29

**Authors:** Tim Tenbensel

**Affiliations:** School of Population Health, University of Auckland, Private Bag 92109, Auckland, 1142 Auckland, New Zealand

It’s a pleasure and a privilege to be invited to address your conference all the way from Auckland New Zealand. I was invited to give this presentation on the basis of a book that I co-edited with my colleague, Kieke Okma. This is a book that traces the experience of health reform across 12 small to medium-sized countries [1]. Our book covers a very broad range of countries, including the Netherlands, New Zealand, Slovenia, Israel, Chile, Tanzania and Taiwan^1^.

My brief for this address was to provide a ‘big picture’ of health reform internationally. In today’s presentation I want to focus mainly on health reform in high-income countries, because the health reform challenges and dynamics of low and middle income countries are very different in many respects. 

The main conclusion of our own book was that health system reform rarely delivers what it sets out to achieve. In this address I seek to explore, at a high level, why this is generally true, despite the enormous variation that exists between health systems. As a political scientist, my interest in understanding health policy goes back to the late 1990s and health policy implementation has been central to this work. My intention is not to promote pessimism, however. It is important to understand where significant health system change comes from, and that might help us harness the forces of change more effectively.

Health systems are difficult to change intentionally. Most of the reasons for this can be traced to the political economy of health. The defining and distinctive feature of health systems (compared to education and social services) is that they are the creation of health professionals, and the most enduring features have their origins in how medical professions in particular collectively organise the delivery of health services [2–4].

The forces of professionalization have three important features that shape health policy and health system reform possibilities. Firstly, the deepening of professional specialization is a significant driver of health system costs - the more specialized a professional is, generally the more training they need and the more they cost [5].

Secondly, all high-income countries have health systems in health services are predominantly financed through ‘third-party payers’ in the form of insurance organisations and governments. Health economists have long described the dynamics of ‘supplier-induced demand’ in which the market relationship between the third-party payer and the provider of services is heavily weighted to the provider because of their superior knowledge base [6].

Thirdly, the whole health sector is defined in terms of scopes of practices about which professions can (and can’t) do particular things. Because the health professional workforce is relatively mobile, this system of scopes of practice has a significant international dimension placing potentially significant limitations on the shape and scope of health reform [7].

A key consequence of all these factors is that health professions (and the medical profession in particular) typically have considerable power over both the formulation and the implementation of any health reform, and there are multiple examples of this dynamic that we cover in our book [8–12].

Compared to many other areas of policy, governments and regulators are often reluctant to shape key features of health systems and services. As an example, the distribution of primary care doctors has major effects on a health system and the health of a population. Yet many governments in high income countries are reluctant to regulate where ambulatory care doctors set up shop [3, 13, 14] .

Over the past four decades there have been many important health policy reform movements that have crossed international borders. I will briefly mention four – but this is by no means a complete list.

In the 1980s and 1990s, the escalating cost of health systems created alarm in most high-income countries, and prompted a very significant wave of reform efforts to control these increases.

In a closely related development around the turn of the millennium, there was a sudden interest in the topic of ‘explicit priority-setting’ in health care in places such as Sweden, New Zealand, the Netherlands and most famously the US state of Oregon [15]. This was the idea that it was not acceptable to allocate health resources blindly, based on historical patterns. There needed to be a more rational, justifiable way of prioritizing health expenditure.

In the twenty first century, governments and health system reformers more generally have emphasized improved integration of health care services to counteract the fragmentation of services, and the fragmented funding systems that patients and their families have to navigate [16].

And most recently, the problem of inequitable health outcomes has risen towards the top of many health reform agendas. The patterns of ‘why some people are healthy and others not’ [17] are now the object of major reforms in my own country, but this is very much an international movement. Each of these movements have risen and sometimes fallen over the past 30 years across many countries.

However, while there are many international health policy reform movements in which problems and solutions seem to be defined in similar ways, this does not mean that all or most high-income countries are heading in the same direction.

In fact, one of the key messages in our book is that health reforms in different countries do not follow a convergent path. When you look into the detail of specific health reforms motivated by these agendas, across different countries, it soon becomes apparent that the details of the problems are quite different, and the solutions are shaped by the design of the health system. This is because these international reform agendas are ‘refracted’ through the prism of each country’s health care system [18].

The most important system feature is how the health system is financed. Insurance-based systems work very differently to health systems financed by taxation [19]. Most of the key decisions in high income countries about the financing of health systems were in place by the 1960s and there has been little change in the pattern since that time.

When we compare health systems, the first thing to look for is the ‘historical bargain’ between governments and the medical profession. If doctors agreed to be paid by the state, for example, they usually demand something significant in return at the time, such as the right to be paid by fee for service. The details of this bargain vary enormously even between countries with similar systems [20, 21].

Political scientists use the terms ‘path-dependence’ [22] and ‘accidental logics’ [4] to describe the influence of health care financing and historical bargains. The idea is that once an initial path is established, it becomes increasing difficult to divert from it.

This means that every health system has quite different ‘stress points’ where conflict and dissatisfaction are most visible. In social insurance systems, the level of premiums that are paid by employers and employees is a typical stress point. In tax-based systems, the stress point is visible rationing via long waiting times for primary care doctors or waiting lists for elective surgery.

In most countries, there is a considerable degree of public dissatisfaction with the health system [23], but the reasons underlying this dissatisfaction vary considerably. That is why the stories of health reform we cover in our book vary widely in their ambitions. Some countries want to increase the role of markets, some want to address market side-effects. In countries that have used market mechanisms in health reform, there have been a wide variety of mechanisms used, and they are used for very different purposes [24, 25].

This means that policymakers need to be very careful in adopting policies and solutions developed in other countries with different histories and paths, even though they might seem to be ostensibly directed at the same problem.

Drawing on a large range of health system reform initiatives covered in our book and elsewhere over the past 30 years very few high-income countries have attempted major reforms in their system of health financing – the major feature that shapes the health system.

Most reforms involve secondary features such as how providers get paid, changes to the organisational design of health system, or regulatory changes to health insurance systems. Nevertheless, many of these reforms were not trivial in their aims.

Kieke Okma and I concluded in our book that ‘ambitious big bang reforms often turned into processes of incremental change that can be characterized, after Charles Lindblom, as “muddling through” [1] (p324). Sometimes this watering down of reform occurs during the policy making process as reform decisions are shaped by compromises. But we also found many examples where reform decisions appeared significant, but were weakened or diverted through implementation processes [9, 11, 12].

And in cases where reforms are not significantly watered down through implementation, we find that the intended effects often do not materialise and the original problem is not solved [8, 10].

For example, the Netherlands embarked on a famous example of health system reform in 2006. The origins go back to the 1980s and were initially inspired by the desire for a system that was better at controlling costs and achieving efficiency. The key mechanism to do this was by creating a market environment in which insurance organisations competed with each other for members. Insurers were also expected to develop different contracting arrangements with providers so that those who successfully contained costs could then compete for members by offering lower premiums.

These reforms had widespread agreement from political parties and key stakeholders, and were largely implemented as designed [24]. But after 15 years, this reform had not resulted in lowering the cost of the health system and may have had the opposite effect. Health spending as a % of GDP rose faster in the Netherlands after 2006 than it did in neighbouring countries [10].

In my second example, New Zealand made major changes to its health system in 2001. These reforms included changing the way that the state paid primary care doctors from fee-for-service to capitation, and the creation of new intermediate organisations to manage this new approach and contract with government funders of health. These changes were made primarily to address significant inequalities in access to primary health care.

In the early years of implementation there was some improvement in access to primary care for many groups [26], but those gains did not persist as government funding of health care tightened during the 2010s. The change to capitation has had little impact on primary care doctors, largely because a significant proportion of their income is still derived from fee-for-service and this continues to dominate their thinking [12].

What these reform stories exemplify is that health systems are very difficult things to steer, and that governments, whatever their role, have limited capacity to turn reform blueprints into desired change.

This sounds quite pessimistic if you are interested in making positive change to a health system. But perhaps there is a different way to think about the topic. Although it is difficult to engineer health system reform, the political economy of health is changing in many ways. There are major shifts emerging in patterns of health need, driven by the increased prevalence of multi-morbidity and complex chronic diseases. Among other effects, COVID-19 has accelerated changes in both the demand for, and design and delivery of mental health services in the community.

Health workforce shortages are common to most if not all high-income countries. Medical professionals cannot do all the things they have done in the past and the dynamics and scopes of practices of different health professions are shifting. After a long lag behind other sectors, we are just starting to see how health information can be used more effectively, and the response to COVID has accelerated that process in many countries, and this can be expected to change power dynamics.

At the local level, there are an increasing number of experiments with new organisational and service models, of which Kinzigtal in Germany [27] and the Canterbury Clinical Network in New Zealand [28] have been prominent examples.

Even though health reforms rarely achieve the results that are intended by policymakers, there may be some unanticipated positive consequences. Because health systems are so path-dependent, reforms have the potential to disrupt ossified structures and practices, and release some new energy into the system. In doing so, major policy reforms may sometimes help to accelerate changes driven by economic, demographic and workforce dynamics.

In New Zealand, for example, one major policy success of the last 30 years has been the creation of the Pharmaceutical Management Authority (known as PHARMAC). This organization was established in the 1990s and it does a very effective job in keeping the costs that the New Zealand government pays for pharmaceuticals very low compared to most countries [29]. PHARMAC was formed because the new organisations created in New Zealand’s 1990s health reforms were creative and saw an opportunity to collectively address the problem of rising drug costs.

However, the consequences of reform-induced energy release may also be quite negative. The perpetual ‘re-disorganisation’ of the English health system since the 1980s has had many profoundly negative consequences [30]. There are certainly some important lessons from countries such as England and New Zealand in which political and health system institutional arrangements enable governments to make frequent major reforms. For those arguing for major reform in countries that are more constrained by path-dependent political and health financing institutions, there are some salutary lessons to be learnt.

Overall I would argue that health system change in high-income countries over the past forty years has far more to do with evolutionary developments characteristic of complex adaptive systems [31], that originate from throughout the system, rather than from a central brain. While this way of thinking about health systems has already begun to influence the way governments actually design health reform, we won’t know for some time whether this will improve the odds of success for ‘intentional’ health system reform.

## Footnotes

1 Zi Health Services Research Congress 2022, September 7 and 8, 2022, Berlin, Germany. The conference website can be found at https://www.zi-congress.de/en/ and the livestream recordings are available at https://www.zi-congress.de/livestream
